# Research on Safety and Compliance of a New Lower Limb Rehabilitation Robot

**DOI:** 10.1155/2017/1523068

**Published:** 2017-07-26

**Authors:** Yongfei Feng, Hongbo Wang, Hao Yan, Xincheng Wang, Zhennan Jin, Luige Vladareanu

**Affiliations:** ^1^Parallel Robot and Mechatronic System Laboratory of Hebei Province, Key Laboratory of Advanced Forging & Stamping Technology and Science of Ministry of Education, Yanshan University, Qinhuangdao 066004, China; ^2^Institute of Solid Mechanics of Romanian Academy, 010141 Bucharest, Romania

## Abstract

The lower limb rehabilitation robot is an application of robotic technology for stroke people with lower limb disabilities. A new applicable and effective sitting/lying lower limb rehabilitation robot (LLR-Ro) is proposed, which has the mechanical limit protection, the electrical limit protection, and the software protection to prevent the patient from the secondary damage. Meanwhile, as a new type of the rehabilitation robots, its hip joint rotation ranges are different in the patient sitting training posture and lying training posture. The mechanical leg of the robot has a variable workspace to work in both training postures. So, if the traditional mechanical limit and the electrical limit cannot be used in the hip joint mechanism design, a follow-up limit is first proposed to improve the compatibility of human-machine motion. Besides, to eliminate the accident interaction force between the patient and LLR-Ro in the process of the passive training, an amendment impedance control strategy based on the position control is proposed to improve the compliance of the LLR-Ro. A simulation experiment and an experiment with a participant show that the passive training of LLR-Ro has compliance.

## 1. Introduction

Cerebral vascular disease, hemiplegic, and paraplegia may cause limb motor dysfunction. Based on the nerve rehabilitation theory, patients could recover through the specialized rehabilitation training [1]. The lower limb rehabilitation robot is an application of robotic technology for people with lower limb disabilities [2]. In recent years, research on the lower limb rehabilitation robots has become an active topic [3, 4]. Several kinds of lower limb rehabilitation robots have been developed. Those can be divided into the trainers with single degree of freedom [5], wearable trainers [6], suspended gait trainers [7, 8], and sitting/lying gait trainers [9]. Because of the specificity of the lower limb rehabilitation robot, it has high demand on the safety and compliance. There is still a lack of widespread randomized clinical trials of the lower limb rehabilitation robots in the hospitals. The safety and compliance have been key issues for robot design and control.

Rehabilitation robotic is the interdisciplinary science. Most of the rehabilitation robots are based on medical rehabilitation principle, modeling on body parameters of normal people, and the lack of the unknown design error, model uncertainty, and parameter self-correction [10]. Based on security considerations, the electrical limit switch is installed on the robots. However, it cannot fully guarantee the patient in the whole training process. Erol and Sarkar used an automatic release rectifier controller on PUMA 560 to provide a quick release of the electromagnet that is fixed on the robot, so as to ensure that the affected limb can be quickly withdrawn from the rehabilitation equipment [11]. Tejima and Stefanov [12] have designed a reflex mechanism that is similar to a biological reflex. When the machine detects unexpected force, it will make a back reaction to prevent collision damage. Some of rehabilitation robots are equipped with special vision-based proximity sensors that perceive the human body and automatically stop the robot when they move too closely toward the user [13]. ViGRR is also to ensure safety by releasing the user from the robot. The footplate is magnetically attached and can be released on demand to mitigate safety risk [14]. The above methods ensure the safety of the patients to some extent. But the realization of these methods is just based on the stability of the electrical system of the rehabilitation robot. When the electrical system is in the unsteady state, the safety of the patient without controlling ability of limb motion may suffer huge risks. So, the mechanical limit protection has to be designed in the rehabilitation robot. However, the sitting/lying lower limb rehabilitation robot as a new type of mechanism [15, 16], its hip joint ranges are different in the sitting training posture and lying training posture. So, the mechanical leg of this type robot has a variable workspace. The traditional mechanical and electrical limit is no longer applicable, and a follow-up mechanical-electrical limit protection is first proposed.

Besides, the mechanical limit and electrical limit protections only aim to prevent the accident from the outside world. They are powerless to deal with the human body's own discomfort and muscle spasm. The interactive force control between robot and patient is a very important aspect in the research of lower limb rehabilitation robot. An interactive control method with active compliance characteristics can avoid the limb and the robot to generate a confrontation, due to the abnormal muscle activity, such as convulsion and tremor. Yang's team controls the upper limb rehabilitation robot by impedance control strategy based on the position, and the control structure is a double-closed loop [17]. Duschau-Wicke et al. used impedance control method to build the virtual wall around the ideal training path to ensure the activity of the lower limb [18]. The impedance control could achieve the dynamic relationship between the force and position and has a good flexibility and security. According to the impedance control strategy based on the position, an amendment impedance control strategy is proposed in this paper to improve the compliance of rehabilitation robot, and its effectiveness is verified by the experiment.

## 2. Materials and Methods

### 2.1. Innovative Design of the LLR-Ro

**Figure 1:**
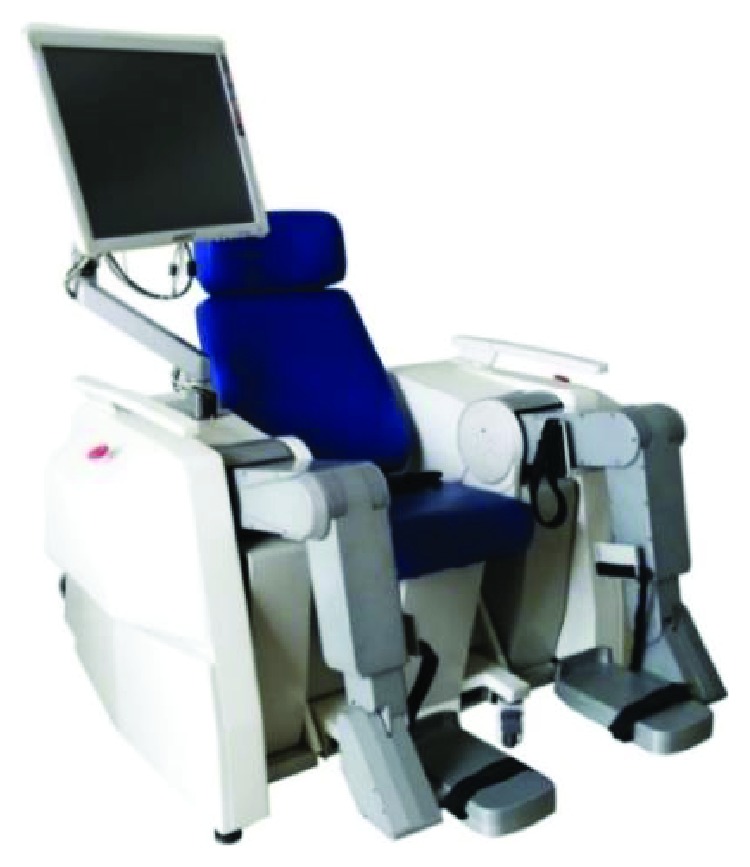
The prototype of the LLR-Ro.

**Figure 2:**
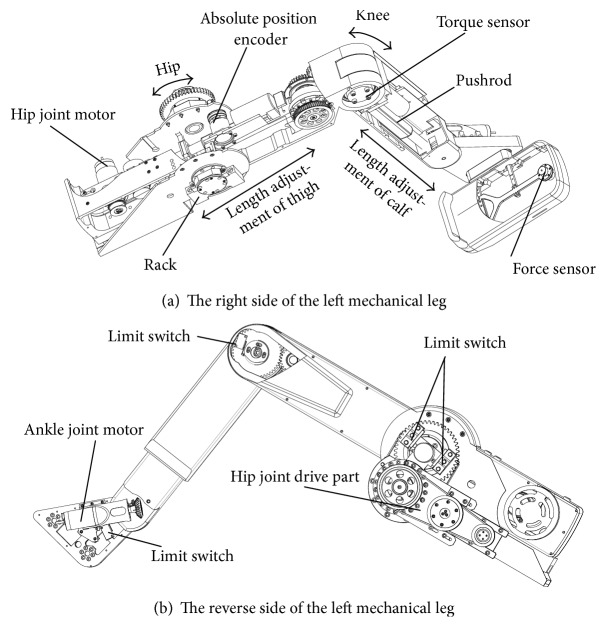
The detailed design of LLR-Ro leg mechanism.

The LLR-Ro contains the movable seat, the left mechanism leg module, the right mechanism leg module, the touch screen, and the control box as shown in Figure 1. The left mechanism leg module and the right mechanism leg module are bilateral symmetry. Each module has a mechanical leg, which has the hip, knee, and ankle joint in the human body sagittal. The mechanical leg is the most important part of the prototype as shown in Figure 2. In order to satisfy people with different height from 1500 mm to 1900 mm, the lengths of the thigh and calf of the LLR-Ro could be changed automatically. The sensing system contains sensors to measure joint rotations and estimate the torque and force produced by the patients. The torque sensors are installed on the location of the joint axis, which can directly acquire joint torque values.

As the LLR-Ro is a typical human-machine system, the limb safety of the patient is the most important principle to be considered in its design. LLR-Ro has the mechanical limit, the electrical limit, and the software protection to prevent the patient from the secondary damage. The design of the mechanical limit mainly takes advantage of the mechanical structure to limit the motion range of joints. Limit switches are installed on extreme position of the hip joint, knee joint, and ankle joint to realize the electrical limit. Patient information will be recorded into the control system during the human-machine interaction. Then, the actuators will control the DC servo motors moving in the motor-designated scope to realize software limit protection.

### 2.2. The Design of the Hardware Control System

**Figure 3:**
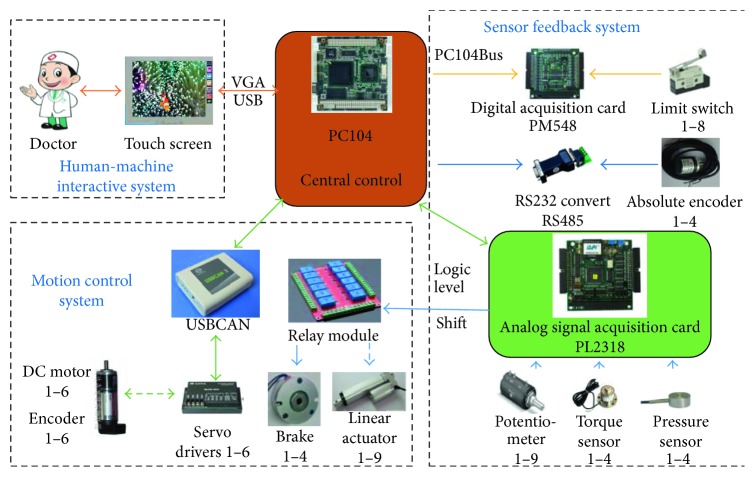
The structure of the hardware control system.

Based on the functions of the LLR-Ro, the hardware control system contains the central control module, the human-machine interactive system, the sensor feedback system, and the motion control system as shown in Figure 3. The central control module mainly runs the control software and receives the operational order from the human-machine interactive system. The human-machine interactive system displays the control software interface and feeds back the training conditions. The motion control system receives the motion control commands from the central control module, realizes the motor closed-loop control and feeds back the joint real motion condition to the central control module. The sensor feedback system acquires the sensor information and achieves the sensor state.

### 2.3. The Follow-Up Limit Design of the LLR-Ro

**Figure 4:**
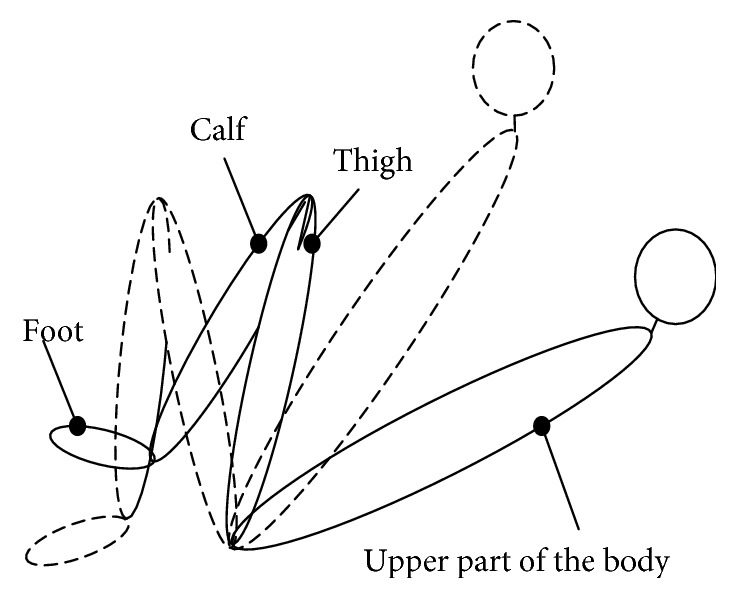
Training posture sketch of the patient.

To meet the needs of the patient with different recovery stages, the back angle of the movable seat could be altered from 110° to 170° to help the patient realize sitting and lying training posture. However, there is a safety angle between the thigh and the upper part of the body in both sitting posture training and lying posture training as shown in Figure 4. If the upper part of the body is at the dotted line position and the thigh is at the full line position, it would bring the patient a secondary damage. So, the mechanical leg in the sitting posture training and the lying posture training has different training workspaces. If the traditional mechanical limit and the electrical limit cannot be used in this hip joint design, then a follow-up limit is proposed to solve this problem.

**Figure 5:**
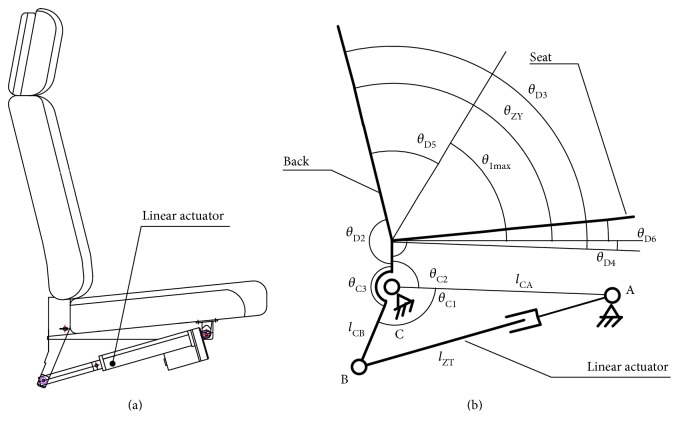
The design of the seat. (a) The structure of the prototype; (b) the simplified model of the seat.

In order to prevent the mechanism leg squeezing in the patient when the seat back angle is adjusted, the angle between the back and the mechanical leg will be limited at 45°~170°. The design of the seat back adjustment takes advantage of the link mechanism with linear actuator as shown in Figure 5(a).  Figure 5(b) shows the simplified model of the seat. *l*_ZT_ is the length of the linear actuator. Point A and point C are fixed on the seat rack. *θ*_C3_,  *θ*_D2_,  *θ*_D4_,  *θ*_D6_,  *l*_CA_, and *l*_CB_ are the constant. *θ*_D5_ equals 45°.

Based on the geometry relationship,

(1) $$\begin{array}{c} \theta_{C1}=\arccos\frac{l_{BC}^{2}+l_{CA}^{2}-l_{ZT}^{2}}{2l_{BC}l_{CA}}\\ \theta_{C2}=360-\theta_{C1}-\theta_{C3}\\ \theta_{D1}=180-\theta_{C2}\\ \theta_{D3}=360-\theta_{D2}-\theta_{D1}. \end{array}$$

The seat back angle could be gotten as

(2) $$\begin{array}{c} \theta_{ZY}=\theta_{D3}-\theta_{D4}, \end{array}$$

and then, the max value of the hip joint would be obtained.

(3) $$\begin{array}{c} \theta_{1\max}=540-\theta_{D2}-\theta_{D4}-\theta_{C3}-\arccos\frac{l_{BC}^{2}+l_{CA}^{2}-l_{ZT}^{2}}{2l_{BC}l_{CA}}-\theta_{D5}. \end{array}$$

Equations (1) and (2) are the relationship between the seat back angle and the linear actuator; (1) and (3) are the relationship between the max value of the hip joint and the linear actuator.

**Figure 6:**
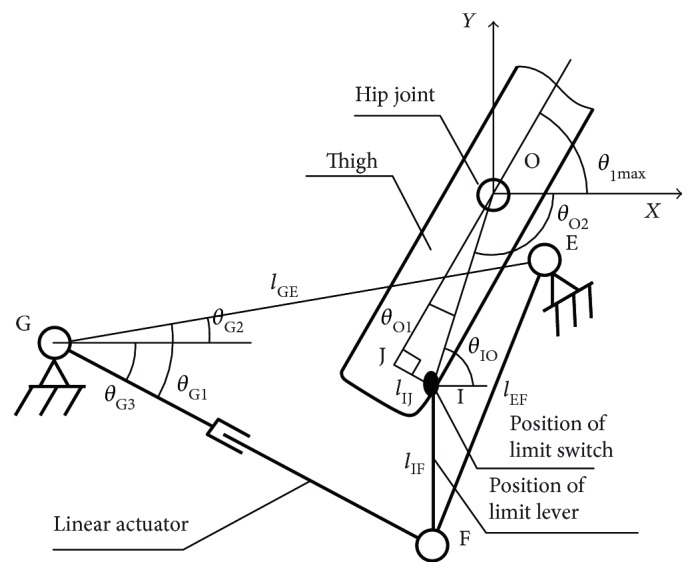
The design of the follow-up limit.

The follow-up limit design of the hip joint mechanism will also adapt the link mechanism with linear actuator as shown in Figure 6. Point O is the center of the hip joint; *l*_XT_ is the length of the linear actuator; point G and point E are fixed on the mechanism rack; and point I is the hip joint limit switch trigger point. The line segment IF represents the mechanical limit rod. *l*_IF_,  *l*_GE_,  *l*_EF_,  *l*_JI_, and *θ*_G2_ are the constant. Coordinate system is established, where O is the origin point, *x* is the horizontal direction, and *y* is the vertical direction.

Based on the geometry relationship,

(4) $$\begin{array}{c} \theta_{G1}=\arccos\frac{l_{XT}^{2}+l_{GE}^{2}-l_{EF}^{2}}{2l_{XT}l_{GE}}\\ \theta_{G3}=\theta_{G1}-\theta_{G2}. \end{array}$$

Then the coordinates of point *I*(*x*_I_,  *y*_I_) could be obtained if

(5) $$\begin{array}{c} x_{I}=x_{G}+l_{XT}\cos\;\theta_{G3}\\ y_{I}=y_{G}-l_{XT}\sin\;\theta_{G3}+l_{IF}. \end{array}$$

The angle *θ*_O1_ could be gotten if

(6) $$\begin{array}{c} \theta_{O1}=\arctan\frac{l_{IJ}}{\sqrt{x_{I}^{2}+y_{I}^{2}}}. \end{array}$$

The relationship between the maximum value of the hip joint angular position with the length of the linear actuator will be calculated.

(7) $$\begin{array}{c} \theta_{1\max}=180-\theta_{O2}-\theta_{O1}=\arctan\frac{y_{F}+l_{IF}}{x_{F}}-\arcsin\frac{l_{JI}}{\sqrt{x_{F}^{2}+{\left(y_{F}+l_{IF}\right)}^{2}}}, \end{array}$$

where

(8) $$\begin{array}{c} x_{F}=x_{G}+l_{XT}\;\cos\left(\arccos\frac{l_{XT}^{2}+l_{GE}^{2}-l_{EF}^{2}}{2l_{XT}l_{GE}}-\theta_{G2}\right)\\ y_{F}=y_{G}-l_{XT}\;\sin\left(\arccos\frac{l_{XT}^{2}+l_{GE}^{2}-l_{EF}^{2}}{2l_{XT}l_{GE}}-\theta_{G2}\right). \end{array}$$

Combining (2), (3), and (7), the relationship between the seat back angle and the length of linear actuator on the follow-up limit device could be achieved. When the seat back angle is changed, the max limit position of hip joint will be calculated.

### 2.4. The Control Compliance of the LLR-Ro

**Figure 7:**
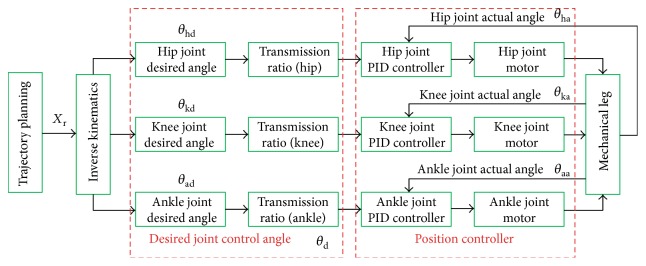
The passive training control based on PID method.

Most of passive training control strategies of the rehabilitation robot are designed in accordance with the strategy of industry robots as shown in Figure 7. In the passive training process, the LLR-Ro drives the patient lower limb along a certain trajectory. However, the passive training is mainly suitable for the patients in the early phase of their illness. At this phase, patient lower limbs have large muscle tone. If the patient feels uncomfortable and his legs move achingly while the mechanism leg of the LLR-Ro still in motion, the patient leg would be hurt again. So, the research on the motion compliance of the LLR-Ro is necessary.

**Figure 8:**
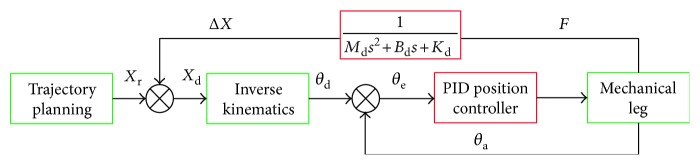
The LLR-Ro impedance control model.

The LLR-Ro also adapts the impedance control strategy based on position control to realize accuracy position control. The impedance control strategy is shown in Figure 8.

The position correction Δ*X* will be obtained through the force *F* in the impedance control model. The impedance control model is introduced by

(9) $$\begin{array}{c} M_{d}\Delta \ddot{X}+B_{d}\Delta \dot{X}+K_{d}\Delta X=F\\ F=F_{PM}+G_{P}+G_{R}, \end{array}$$

where *M*_d_,  *B*_d_,  and *K*_d_ are the target inertia matrix, the damping matrix, and the stiffness matrix, respectively; Δ*X* is the position correction of the mechanism leg; *F*_PM_ is the contact force from the patient muscle tone; and *G*_P_ and *G*_R_ represent the weight from the patient lower limb and LLR-Ro mechanical leg, respectively.

It can be obtained through the Russ transform:

(10) $$\begin{array}{c} \Delta X\left(s\right)=\frac{F\left(s\right)}{M_{d}s^{2}+B_{d}s+K_{d}}. \end{array}$$

**Figure 9:**
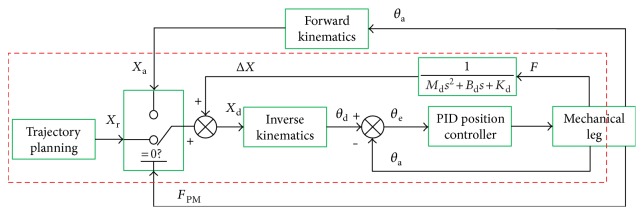
The amendment impedance control strategy.

When the patient feels uncomfortable and has no ability to move with the robot along the trajectory during the training, the contact force *F*_PM_ from the patient muscle tone will be bigger. Based on the above impedance control strategy to realize the compliance in the motion along the preplan trajectory, we also need to change the end point force to position the adjustment amount through the impedance control model. The impedance control strategy will be modified to the new amendment impedance control strategy to suit the rehabilitation training as shown in Figure 9. This new control strategy contains two parts, including the traditional impedance control loop in the dashed frame and the opposite direction correction control loop out of the dashed frame. The two parts are connected through a switch block.

When the force *F*_PM_ equals zero, the amendment impedance control strategy is just as the common impedance control strategy to make the robot move along the preplan trajectory. If the force *F*_PM_ does not equal to zero, the opposite direction correction control loop is closed. The contact force will be converted to position correction through the impedance control model. The actual position *X*_a_ adds the position correction Δ*X* to get the amendment trajectory *X*_d_. The LLR-Ro moves along the amendment trajectory until the contact force *F*_PM_ equals to zero, then the LLR-Ro shuts down. The physician will check the patient security to determine the next training program.

### 2.5. Approximate Calculation of the Force from Patient Accident Force

**Figure 10:**
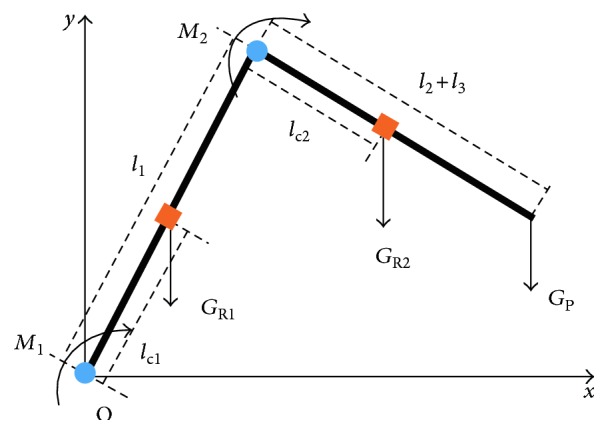
The statics model to calculated the torques.

As the length of the foot is very short relative to the calf and thigh, in the passive training, the ankle joint moves in a small motion range and it will be planned separately. In this paper, the ankle joint of LLR-Ro mechanical leg is fixed. There are torque sensors installed on the hip joint and knee joint of the mechanical leg to measure the LLR-Ro joint torques. The measured torques contain three parts, *F*_PM_, *G*_P_, and *G*_R_. Because of the length adjustment of the mechanical leg and the transmission parts installed on the mechanical leg with a nonuniform distribution, the position of the centroid is difficult to determine. So, it cannot use the dynamics directly to calculate the torques generated by the weight of the mechanism leg. However, the LLR-Ro moves in a low velocity and steady state, and the effect from dynamics can be ignored. The torques generated by the weights of the mechanical leg and patient leg will be calculated through the statics. The statics model is shown in Figure 10. *G*_R1_ represents the weight of the mechanical leg thigh, and *G*_R2_ represents the weight of the mechanical leg calf and foot, respectively. *l*_c1_ and *l*_c2_ represent the distance from the spindle to the calf plus foot centroid and thigh centroid, respectively. The knee torque generated from the weight of LLR-Ro mechanical leg calf as well as patient leg and the hip torque generated from the weight of mechanical leg as well as patient leg could be calculated through the joint torque sensors, respectively.

(11) $$\begin{array}{c} M_{2z}=G_{R2}l_{c2}\;\cos\left(\theta_{1}+\theta_{2}\right)+G_{p}l_{2}\;\cos\left(\theta_{1}+\theta_{2}\right)=B\;\cos\left(\theta_{1}+\theta_{2}\right)\\ M_{1z}=G_{R1}l_{c1}\;\cos\;\theta_{1}+\left(G_{R2}+G_{p}\right)l_{1}\;\cos\;\theta_{1}+M_{2}=A\;\cos\;\theta_{1}+M_{2}. \end{array}$$

The values of *A* and *B* will be changed when the length of the mechanical leg is varied. It could be calculated through the experiment. When the patient exerts force *F*_PM_ on the end of mechanism leg, the torques *M*_1P_ and *M*_2P_ would be generated at the hip joint and knee joint. *F*_PM_ could be seen as a component force from *F*_*x*_ and *F*_*y*_.

*F*_*x*_ and *F*_*y*_ are the forces along the horizontal direction and vertical direction.

(12) $$\begin{array}{c} \left[\begin{array}{c} M_{1P}\\ M_{2P} \end{array}\right]=\left[\begin{array}{cc} -l_{1}\sin\;\theta_{1}-l_{2}\;\sin\left(\theta_{1}+\theta_{2}\right) & l_{1}\cos\;\theta_{1}+l_{2}\;\cos\left(\theta_{1}+\theta_{2}\right)\\ -l_{2}\;\sin\left(\theta_{1}+\theta_{2}\right) & l_{2}\;\cos\left(\theta_{1}+\theta_{2}\right) \end{array}\right]\left[\begin{array}{c} F_{x}\\ F_{y} \end{array}\right]=J^{T}\left(q\right)\left[\begin{array}{c} F_{x}\\ F_{y} \end{array}\right], \end{array}$$

where *J*^T^(*q*) is the Jacobin matrix transposition.

The contact force could be calculated as follows:

(13) $$\begin{array}{c} F_{PM}=\left[\begin{array}{c} F_{x}\\ F_{y} \end{array}\right]={\left(J^{T}\left(q\right)\right)}^{-1}\left[\begin{array}{c} M_{1P}\\ M_{2P} \end{array}\right]={\left(J^{-1}\left(q\right)\right)}^{T}\left[\begin{array}{c} M_{1P}\\ M_{2P} \end{array}\right], \end{array}$$

where

(14) $$\begin{array}{c} {\left(J^{-1}\left(q\right)\right)}^{T}=\frac{1}{l_{1}l_{2}\;\sin\;\theta_{2}}\left[\begin{array}{l} l_{2}\;\cos\left(\theta_{1}+\theta_{2}\right)-l_{1}\;\cos\;\theta_{1}-l_{2}\;\cos\left(\theta_{1}+\theta_{2}\right)\\ l_{2}\;\sin\left(\theta_{1}+\theta_{2}\right)-l_{1}\;\sin\;\theta_{1}-l_{2}\;\sin\left(\theta_{1}+\theta_{2}\right) \end{array}\right]. \end{array}$$

At this time, the actual torque of the hip joint *M*_1t_ and the actual torque of the knee joint *M*_2t_ are measured by the torque sensors, and we could get the torques exerted by the patient as follows:

(15) $$\begin{array}{c} M_{1P}=M_{1t}-M_{1z}\\ M_{2P}=M_{2t}-M_{2z}. \end{array}$$

At last, the contact force *F*_PM_ from the patient muscle tone could be calculated through (13) and (15).

## 3. Results

### 3.1. The Functional Verification Experiment of the Follow-Up Limit

Based on the mechanical design of the movable seat and mechanical leg rack, the geometric parameters in Section 2.3 are given in Table 1. The follow-up limit includes the mechanical limit and electrical limit protections. Both limit protections have the same limit position. When the mechanical leg reaches the hip joint maximum limit position, the limit switch will change its signal and stop the mechanical leg moving. According to the range of the seat back angle, the hip joint maximum limit position could be changed from 65° to 125°. However, the hip joint maximum position is designed at 80°. So, the hip joint maximum limit position is designed from 65° to 80°.

**Figure 11:**
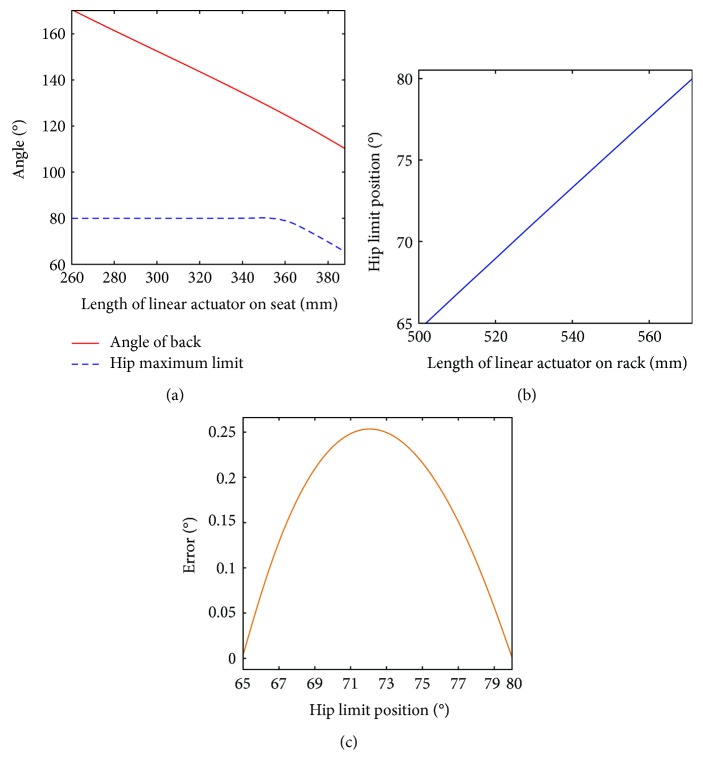
Results of the follow-up limit experiment. (a) The relationship between length of linear actuator on seat and angle of seat back as well as hip maximum limit position; (b) the relationship between length of linear actuator on rack and actual hip maximum limit position; (c) the error of hip maximum limit position.

Based on (3) and (7), when the linear actuator *l*_ZT_ on the seat is changed, the linear actuator *l*_XT_ on the mechanical leg rack is varied correspondingly. The hip joint maximum limit position is measured through angulometer. The results are recorded in Figure 11.  Figure 11(a) shows the relationship between length of linear actuator on seat and angle of seat back as well as theoretical hip maximum limit position.  Figure 11(b) shows the relationship between length of linear actuator on rack and actual hip maximum limit position.  Figure 11(c) shows the error of hip maximum limit position.

### 3.2. Simulation Experiment of the Amendment Impedance Control Strategy

**Figure 12:**
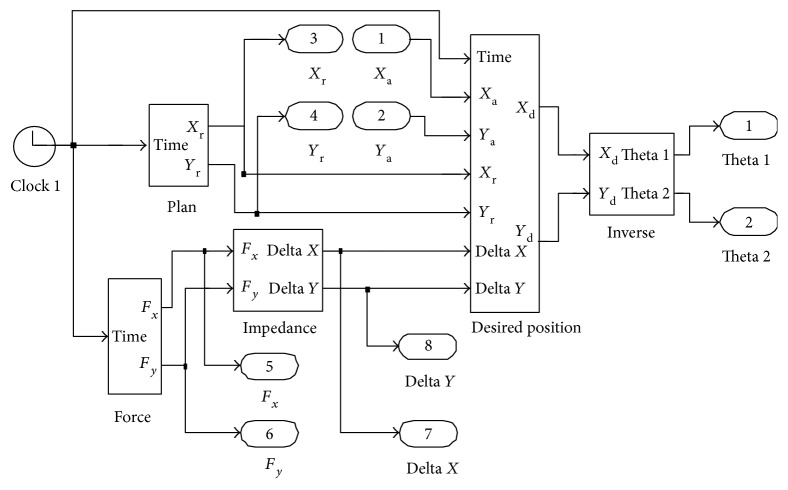
The simulation model of the amendment impedance control strategy.

The theoretical simulation has been researched through MATLAB as shown in Figure 12. In this simulation, the values of *G*_P_ and *G*_R_ are designed as zero. *F*_PM_ is seen as a component force from *F*_*x*_ and *F*_*y*_. *F*_*x*_ and *F*_*y*_ are supposed to be given as below:

(16) $$\begin{array}{c} F_{x}=\left\{\begin{array}{l} 0\;t<3s\\ \sin\;t+0.3\;\sin\;2t-0.2\;\sin\;4t\;3s\leq t\leq 4.5s\\ 0\;4.5s<t, \end{array}\right. \end{array}$$

(17) $$\begin{array}{c} F_{y}=\left\{\begin{array}{l} 0\;t<3s\\ \cos\;t+0.4\;\cos\;3t-0.3\;\cos\;5t\;3s\leq t\leq 4.5s\\ 0\;4.5s<t. \end{array}\right. \end{array}$$

The inputs of the force block are from (16) and (17). Plan block is simulated to get the training trajectory. The impedance block could realize the force transferred as position correction. The inputs of desired position block are from the outputs of plan block and impedance block. The function of inverse block is designed to get each joint position to drive the motors. In this simulation experiment, plan block is the input of a linear trajectory. The positions of the start point and end point are (362.16, 131.36) and (758.69, −30.66), respectively. The parameters *M*_d_,  *B*_d_,  and *K*_d_ are given as follows:

(18) $$\begin{array}{c} M_{d}=\left[\begin{array}{c} 0.0625\\ 0.0625 \end{array}\right],B_{d}=\left[\begin{array}{c} 5\\ 5 \end{array}\right],K_{d}=\left[\begin{array}{c} 100\\ 100 \end{array}\right]. \end{array}$$

**Figure 13:**
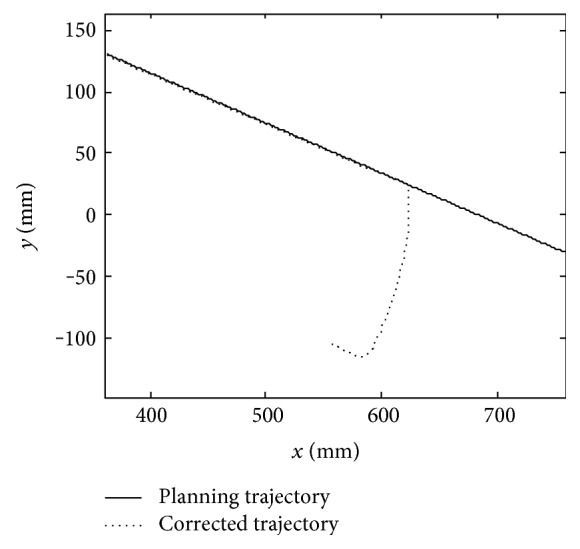
The planning trajectory and trajectory after corrected.

**Figure 14:**
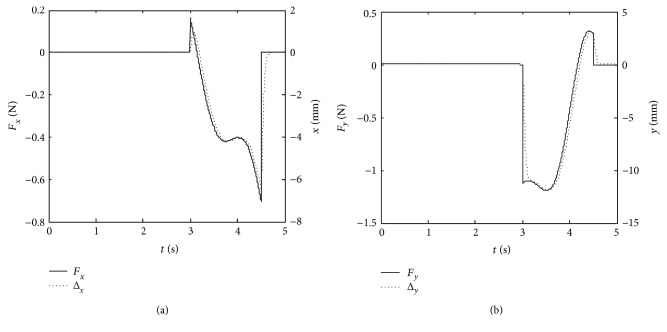
The simulation comparison between the contact force and the position correction. (a) The curves of the force *F*_*x*_ and the correction value along *x*-axis Δ*x*; (b) the curves of the force *F*_*y*_ and the correction value along *y*-axis Δ*y*.

The simulation planning trajectory and corrected trajectory are obtained as shown in Figure 13. The curves of the forces *F*_*x*_ and *F*_*y*_ and the correction values along *x*-axis Δ*x* and *y*-axis Δ*y* are obtained as shown in Figure 14.

### 3.3. Preliminary Experiment of the Amendment Impedance Control Strategy

**Figure 15:**
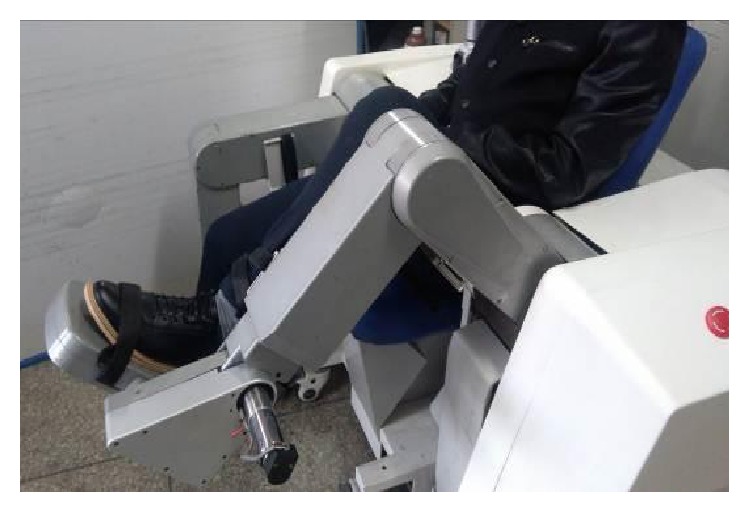
The process of the participant doing the passive rehabilitation training.

In order to verify the innovative design of the prototype and the amendment impedance control strategy, a preliminary experiment is conducted. The experiment is held at the Intelligent Rehabilitation Robot Laboratory in Yanshan University. Before the experiment, approval for all studies is obtained from Yanshan University Ethics Committee, and all the experiment information is introduced to the participant and an informed consent form is signed by the participant. The participant in this experiment is a healthy person, and his left leg is put on the mechanical leg of the LLR-Ro as shown in Figure 15. The participant thigh length *l*_1_ = 350 mm and the calf length *l*_2_ = 350 mm. At the start of the rehabilitation training, the participant is in a relaxed state moving with the LLR-Ro. Based on (11), the values of *A* and *B* are obtained 15.47 N·m and 17.43 N·m, respectively.

Then, the participant force on the mechanism leg suddenly to imitate the participant at uncomfortable state. The software will calculate the contact force through the torque sensors on the mechanism leg. The contact force would be transformed from the joint torques. If the force along the *x*-axis or *y*-axis is beyond (−2N, 2N), the LLR-Ro will be in the amendment impedance control strategy based on the position control to adjust the end trajectory until the contact force is back in (−2N, 2N) and the mechanism leg stops.

The impedance model is applied on the digital control with data discretization. When *m* = 0.0625,  *b* = 5,  and *k* = 200, the transfer function is discretized through the zero-order holder. Sampling time is 150 ms, and the discrete time transfer function could be obtained as

(19) $$\begin{array}{c} G\left(z\right)=\frac{0.00499z+7.728e-06}{z^{2}-0.000413z+4.264e-08}. \end{array}$$

The difference equation could be gotten as

(20) $$\begin{array}{c} x\left(k\right)=0.004958x\left(k-1\right)-0.000006144x\left(k-2\right)+0.009826f\left(k-1\right)+0.000124f\left(k-2\right). \end{array}$$

**Figure 16:**
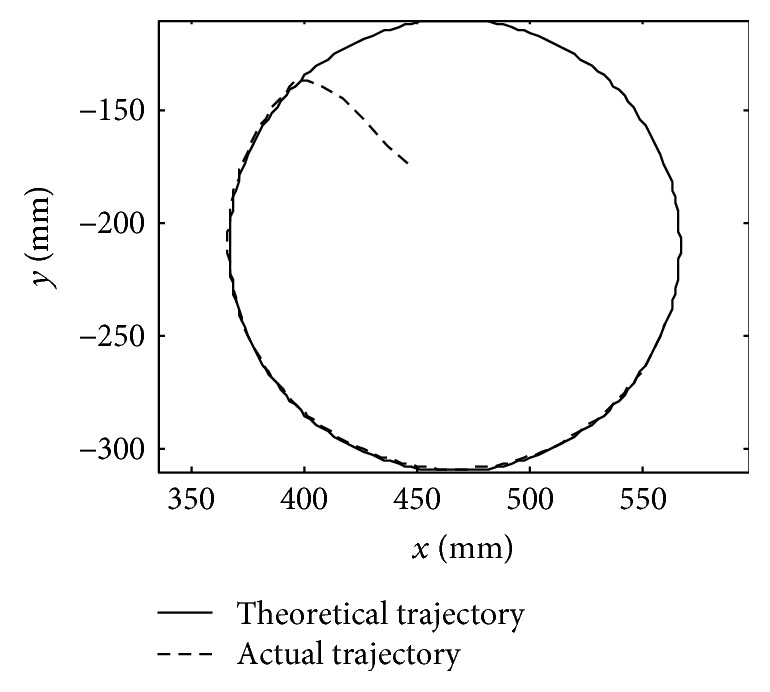
The theoretical and actual motion curves of the mechanism leg end point.

The initial values are *x*(1) = *x*(2) = 0, *f*(1) = *f*(2) = 0. The experiment will use a circle trajectory. The center is (*x*_0_, *y*_0_) = (466.48, −209.83) as shown in Figure 16, and the radius is *r* = 100 mm.

**Figure 17:**
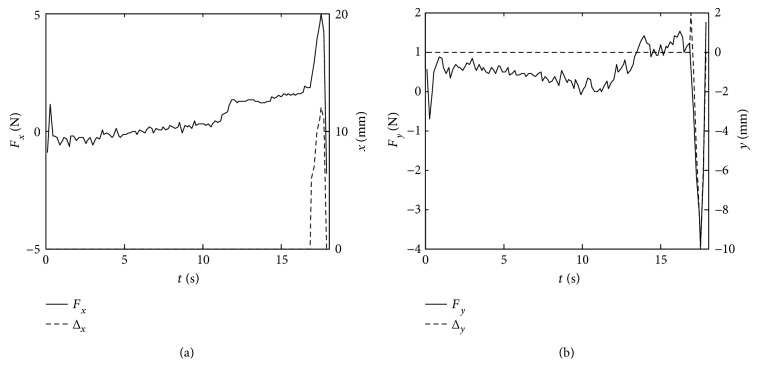
The comparison between the contact force and the position correction. (a) The comparison between the contact force and the position correction along *x*-axis; (b) the comparison between the contact force and the position correction along *y*-axis.

From the experiment, we could obtain that the force along *x*-axis and the position correction versus time and the force along *y*-axis and the position correction versus time are shown in Figure 17.

## 4. Discussion

The follow-up limit experiment is conducted. Figure 11 shows that when the angle of seat back is changed, the hip maximum limit position will be varied accordingly. Although there is an error between the actual hip maximum limit position and the theoretical hip maximum limit position, the largest error is 0.25° and could be acceptable. The design of follow-up limit is feasible. However, this experiment just used limit switch and conducted with a small hip joint speed. Maybe the fast hip joint speed would destroy the linear actuator on the rack. The destructive experiment of the follow-up limit will be verified in the future.

It is necessary to conduct a simulation experiment before the experiment with human participation. From Figures 13 and 14, the curves of correction values along *x*-axis and *y*-axis have the same changing trend with the forces *F*_*x*_ and *F*_*y*_. In the first three seconds, the forces *F*_*x*_ and *F*_*y*_ equal to zero, the position correction values are zero, and the mechanical leg end point moves along the circular trajectory. During the time at 3 s~4.5 s, the forces vary below and beyond zero, the position corrections can follow the variations of forces very fast, and the mechanical leg end moves along the correctional trajectory. When the time is at 4.5 s~5 s, the position corrections turn into zero as the forces *F*_*x*_ and *F*_*y*_ are reducing to zero. Then, the mechanical leg end stops moving. The above simulation process shows that when the patient accident forces emerge, LLR-Ro will decrease the forces through position corrections of the mechanical leg end. This control strategy makes LLR-Ro realize good compliance to make the passive training safe.

The environments of the simulation experiment and an experiment with a participant are a little different. The simulation one ignores the weights from patient leg and mechanical leg. Besides, the simulation experiment also neglects the errors from the mechanical leg assemble and sensor errors. The improvement of the experiment with a participant is considering the errors, and patient muscular tone *F*_PM_ is limited at (−2N, 2N). If *F*_PM_ is beyond the limited range, LLR-Ro will generate position corrections.

From Figures 16 and 17, when the force *F*_PM_ is in the allowable range, the mechanism leg moves along the preplan trajectory. However, when it is beyond the allowable range, the mechanism leg will do the amendment impedance control based on the position. The results are almost same with the simulation experiment. From the experiment results, the amendment impedance control strategy is feasible. From Figure 15, the LLR-Ro could help the volunteer realize passive training. It proves that the mechanism design of the LLR-Ro is feasible and safe.

In the future, the authors will do the research on the active training based on the impedance control strategy, the clinic verification of the LLR-Ro, and biocooperative control strategy. The rehabilitation training patterns are automatically achieved through the intelligent human-machine-cooperative control system. Applying high-tech methods like biomechanical information and patient physiological information, the evaluation system of rehabilitation efficacy of LLR-Ro will be established. Based on the patient training state, LLR-Ro could changed the training parameters by itself during one rehabilitation training pattern.

## 5. Conclusions

A new intelligent lower limb rehabilitation robot (LLR-Ro) is proposed. It can help patients recover lower limb disabilities. Because of the specificity of the lower limb rehabilitation robot, the safety and compliance are researched. The LLR-Ro has a variable workspace. If the traditional mechanical limit and the electrical limit cannot be used in the design, then a follow-up limit is proposed to solve this problem. To prevent the patient from the secondary damage in the passive training, an amendment impedance control strategy based on the position control is proposed to improve the compliance of the LLR-Ro. The simulation experiment and experiment with a participant verify that the control strategy is feasible and the mechanical design of LLR-Ro is safe.

## Figures and Tables

**Table 1:** Geometric parameters of the movable seat and mechanical leg rack.

Name	Geometric parameters		
Movable seat	*θ*_C3_ = 157°	*θ*_D2_ = 166°	*θ*_D4_ = −3°
	*θ*_D5_ = 45°	*l*_CA_ = 324.7 mm	*l*_CB_ = 129 mm

Mechanical leg rack	(*x*_G_, *y*_G_) = (−560 mm, −235 mm)	(*x*_E_, *y*_E_) = (64 mm, −81 mm)	*l*_EF_ = 350 mm
	*l*_IF_ = 150 mm	*l*_IJ_ = 27.5 mm	—
